# Route, origin & valence matter: towards sophisticated next-generation vaccines to cope with the COVID-19 pandemic

**DOI:** 10.1038/s41392-022-01053-4

**Published:** 2022-06-15

**Authors:** Julia Volckmar, Lars Melcher, Dunja Bruder

**Affiliations:** 1 Infection Immunology Group, Institute of Medical Microbiology, Infection Prevention and Control, Health Campus Immunology, Infectiology and Inflammation, Otto-von-Guericke University Magdeburg, Magdeburg, Germany; 2 Immune Regulation Group, Helmholtz Centre for Infection Research, Braunschweig, Germany

**Keywords:** Infectious diseases, Vaccines

In a recent study published in *Cell*, Afkhami and colleagues systematically compared different routes of vaccine delivery, origin of the vaccine platform as well as valence of the vaccine and demonstrated that the respiratory mucosal delivery of a trivalent chimpanzee’s adenovirus (Ad)-vectored vaccine is superior to any other of the tested conditions in inducing broadly-acting immunity and protection against current severe acute respiratory syndrome coronavirus 2 (SARS-CoV-2) and possibly future variants of concern (VOC).^1^

The ongoing global coronavirus disease 2019 (COVID-19) pandemic caused by SARS-CoV-2 has forced an uniquely fast development of novel mRNA- and vector-based vaccines. While these vaccines have proven effective for the control of COVID-19 caused by the ancestral SARS-CoV-2 strain, different VOC escape vaccine-induced immunity resulting in limited protection efficacy against mild to moderate COVID-19. Therefore, there is an urgent need for the development of improved next-generation vaccines.^2,3^

**Fig. 1:**
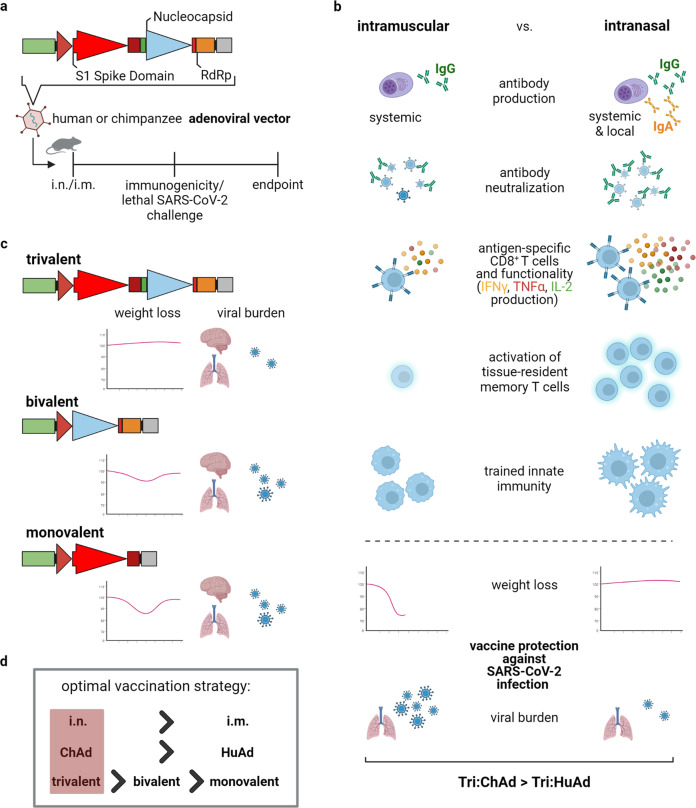
Mucosal immunization with a trivalent ChAd-vectored vaccine induces superior protection against ancestral SARS-CoV-2 and variants of concern. **a** Three different SARS-CoV-2 antigen domains (S1 Spike Protein (S1), nucleocapsid (N), RNA-dependent RNA polymerase (RdRp)) were cloned into either human (Tri:HuAd) or chimpanzee (Tri:ChAd) adenoviral vectors. BALB/c mice were immunized either intranasal (i.n.) or intramuscular (i.m.) prior to lethal SARS-CoV-2 challenge. **b** Results of the comparative analyses utilizing both the two different vector platforms and routes of application (i.n. vs. i.m.). Mucosal immunization (i.n.) with the Tri:ChAd vaccine induced overall superior immune responses: increased production of systemic and local IgG and IgA antibodies, markedly enhanced antibody neutralization capacity, a strong induction of multifunctional CD8^+^ T cell responses as well as the strong activation of T_RM_ and TII. I.n. vaccinated mice infected with an otherwise lethal dose of SARS-CoV-2 showed no body weight loss and a significant decrease of viral burden. **c** Schematic representation of the three different chimpanzee adenoviral constructs: the trivalent (S1, N, RdRp), bivalent (N, RdRp) and monovalent (S1) vaccines. Mucosal delivery of the trivalent vaccine exhibits highest protective efficacy against SARS-CoV-2 and varianst of concern (VOC) indicated by prevention of morbidity (body weight loss) and a significant decrease in viral burden compared to mice that received the bivalent or monovalent vaccines. **d** The optimal vaccination strategy against both SARS-CoV-2 and VOC was proven to be an intranasal application of a trivalent vaccine using the chimpanzee adenoviral vector platform. Created with BioRender.com

Afkhami and colleagues^1^ developed trivalent vaccine candidates expressing the SARS-CoV-2 antigens spike protein 1 (S1), nucleocapsid and the RNA-dependent RNA polymerase (RdRp) protein in adenoviral vectors of human (Tri:HuAd) or chimpanzee (Tri:ChAd) origin (Fig. 1a). Interestingly, both the quantity and quality of the humoral and cellular immune response induced upon vaccination clearly depends on the delivery route. More specifically, a single-dose intranasal immunization with Tri:HuAd or Tri:ChAd was clearly superior in inducing systemic (serum IgG) and mucosal (airway IgG, IgA) neutralizing antibodies compared to intramuscular vaccine delivery (Fig. 1b). Next to the obvious advantage of mucosal vaccination in terms of inducing neutralizing antibody responses in the airways, thorough characterization of the mucosal T cell responses uncovered another weakness of intramuscular compared to mucosal vaccine administration. Irrespective of the vaccine vector used, systemic application failed to induce antigen-specific CD4^+^ and CD8^+^ T cells as well as tissue-resident memory T cells (T_RM_) in the airways. In contrast, mucosal vaccine delivery proved to be highly effective in inducing IFNγ-producing CD4^+^ and multifunctional CD8^+^ T cells, indicated by the high frequency of IFNγ, TNFα and/or IL-2 cytokine producers with cytotoxic potential in addition to the induction of durable multifunctional T_RM_ in the airways (Fig. 1b).

While most studies addressing vaccine efficacy exclusively focus on antibody and T cell responses, Afkhami and coworkers also included the dependency of induced trained innate immunity (TII) on the route of vaccine delivery in their survey. More specifically, they characterized the trained MHC II^high^ alveolar macrophages and interstitial macrophages, and demonstrated that only mucosal, but not systemic, immunization resulted in the strong induction of both macrophage subsets. Remarkably, the vaccine based on the chimpanzee origin (Tri:ChAd) was clearly superior in inducing neutralizing antibodies, T_RM_ and TII compared to the Ad-vector of human origin (Tri:HuAd). Thus, the authors unequivocally demonstrated the outperforming nature of the mucosal delivery route in combination with the chimpanzee-derived Ad-vector vaccine platform.

To ultimately prove that the multilayered immune response induced by mucosal delivery of Tri:ChAd would confer highest protection against fatal SARS-CoV-2 infection, Afkhami and colleagues vaccinated mice either intranasal or intramuscular with a single-dose Tri:HuAd or Tri:ChAd, respectively, followed by a lethal challenge with a mouse-adapted SARS-CoV-2 virus. Indeed, in line with the superior induction of humoral, cellular and trained innate immunity, the outstanding features of the mucosal, but not systemic, applied Tri:ChAd vaccine translated into full protection against an otherwise lethal infection.^1^ Importantly, in contrast to first-generation COVID-19 vaccines which were less effective against immune-evasive VOC,^2,3^ mucosal vaccination with the Tri:ChAd vaccine conferred potent protection not only against lethal challenge with the wild-type ancestral SARS-CoV-2 strain, but as well against the VOC B1.1.7 and B.1.351.

Next to the delivery route and the origin of the Ad-vector vaccine, the valence of the vaccine might be decisive for vaccine efficacy. To experimentally prove this, the authors constructed in addition to the trivalent vaccine the bivalent vaccine Bi:ChAd (nucleocapsid and RdRp) and the monovalent vaccine Mono:ChAd (S1) (Fig. 1c). Mucosal immunization with mono-, bi- and trivalent chimpanzee-derived Ad-vector vaccine and subsequent lethal SARS-CoV-2 challenge revealed morbidity and extensive lung pathology in mice vaccinated with Mono:ChAd. In contrast, Bi:ChAd vaccinated animals appeared clinically stable, but the trivalent vaccine proved the most effective in conferring protection against a severe course of SARS-CoV-2 infection (Fig. 1d). The fact that the Bi:ChAd vaccine was superior than the S1-expressing Mono:ChAd might be simply the consequence of a wider T cell immunity induced by Bi:ChAd against the virus or it might reflect that antigens other than S1 are more effective in inducing SARS-CoV-2-specific cellular immunity. Nevertheless, the study lacks a comparison between different Bi:ChAd vaccines including the S1 antigen that would allow answering this question.

In conclusion, considering many different facets of vaccine design, Afkhami and coworkers made several important key findings that should be considered as blueprint for the development of sophisticated next-generation vaccines. They identified mucosal delivery of a trivalent vaccine incorporated in a chimpanzee-derived Ad-vector as the ideal tool for the stimulation of systemic and, even more important, mucosal antibody and T cell responses together conferring protection against an otherwise devastating SARS-CoV-2 infection.^1^ Without any doubt, the worldwide concerted effort that resulted in the incredibly fast development of first-generation SARS-CoV-2 vaccines was a great success in containing the global pandemics. However, since we are facing the problem that the virus is continuously evolving and thereby capable of escaping immunity induced by current vaccines, the development of improved next-generation vaccines taking into account recent research data is imperative.^2^ Notably, authorized first-generation SARS-CoV-2 vaccines (i.e. mRNA and vector vaccines) clearly differ from the ideal vaccine described by Afkhami *et al*. as they are generally applied intramuscular and are monovalent.^3,4^ While e.g. the ChAdOx1nCov-19 vaccine from AstraZeneca actually is based on a vector system of chimpanzee origin, it is still monovalent and delivered intramuscular, thus offering potential for further optimization.^2,5^ Indeed, there is growing effort in probing the applicability of first-generation COVID-19 vaccines for respiratory mucosal delivery. However, most of the studies published so far did not distinguish between mucosal and systemic delivery routes, nor did they include different VOC.^4,5^ Therefore, the study by Afkhami et al. highlighted here should be considered as a milestone for future vaccine development since it unequivocally demonstrates the superiority of mucosal delivery of a trivalent ChAd-vectored vaccine in protection against ancestral SARS-CoV-2 and VOC.
